# Chronic Copper Bilysinate Poisoning in Five Texel Sheep: A Case Report

**DOI:** 10.3390/life14111363

**Published:** 2024-10-24

**Authors:** Dalma Pivariu, Adrian Nechita Oros, Alexandru Tabaran, Francesca Caloni, Pompei Bolfa, Andras-Laszlo Nagy

**Affiliations:** 1 Department of Toxicology, Faculty of Veterinary Medicine, University of Agricultural Sciences and Veterinary Medicine Cluj-Napoca, Calea Manastur 3-5, 400372 Cluj-Napoca, Romania; dalma.pivariu@usamvcluj.ro (D.P.); orosnadrian@yahoo.com (A.N.O.); 2 Department of Pathology, Faculty of Veterinary Medicine, University of Agricultural Sciences and Veterinary Medicine Cluj-Napoca, Calea Manastur 3-5, 400372 Cluj-Napoca, Romania; 3 Department of Environmental Science and Policy (ESP), Universita degli Studi di Milano, Via Celoria 10, 20133 Milan, Italy; francesca.caloni@unimi.it; 4 Department of Biomedical Sciences, Ross University School of Veterinary Medicine, Basseterre P.O. Box 334, Saint Kitts and Nevis; pompeibolfa@gmail.com (P.B.); anagy@rossvet.edu.kn (A.-L.N.)

**Keywords:** hemolysis, trace minerals, potentially toxic element, toxicity

## Abstract

Copper is an essential trace element but becomes toxic in overexposed animals. Sheep are the domestic species most prone to chronic copper poisoning, as a slight increase in the dietary concentration can lead to liver accumulation and the development of clinical signs in this species. Common sources of copper in the diet are feed additives and mineral supplements, which are commonly used in pigs and poultry. Recently, new copper supplements were registered for animal nutrition, including copper bilysinate. This study describes an episode of presumed chronic copper poisoning in Five Texel sheep, which were exposed to a compound feed containing copper bilysinate. Four weeks after the introduction of the compound feed into the diet, the first animal started to show typical clinical signs of chronic copper poisoning and died, followed by another animal a week later. Despite removing the compound feed from the diet, a third sheep died 3 weeks later. Two animals survived and fully recovered. Necropsy and histology showed characteristic gross and microscopical lesions typical of copper poisoning. The case report highlights the potential toxic effect of copper bilysinate in sheep.

## 1. Introduction

Copper (Cu) is an essential trace element for both ruminants and monogastrics [1] and plays an important role in many physiological processes, as it is a component of proteins, enzymes, and nucleic acids [2,3,4,5,6]. As a component of essential enzymatic systems, copper is involved in energy production, immune response, growth, nervous system function, and connective tissue formation [1,5].

Copper toxicosis is more common in sheep than in cattle or monogastric animals [1], causing important economic losses [7]. Cattle and certain breeds of dogs, notably Bedlington Terriers, are also sensitive. In Bedlington Terriers, an inherited sensitivity to copper toxicosis similar to Wilson disease in humans has been identified. Chronic copper poisoning has been reported in other breeds of dogs, including Labrador Retrievers, West Highland White Terriers, Skye Terriers, Keeshonds, American Cocker Spaniels, and Doberman Pinschers [8,9]. Other species, such as pigs, poultry, and horses, may be affected but are more resistant [7].

The high susceptibility of sheep to copper toxicosis is due to their low dietary requirement and their inability to increase copper excretion in response to increased dietary intake [6]. Certain breeds of sheep are more sensitive than others, Texel sheep being considered one of the most sensitive breeds to copper toxicosis [6]. Milk sheep in intensive farming [7] and young animals are more prone to copper poisoning than adult ones [6]. This age-related susceptibility may be attributable to their higher copper absorption capacity than in adult sheep and their underdeveloped biliary excretion [10].

In sheep, when the diet contains elevated copper levels, or is deficient in molybdenum, copper accumulates in the liver [1]. The most important sources of copper in the diet are feed additives such as copper sulfate, copper chloride, and copper oxide [9]. The use of trace mineralized salt in sheep diets or forage harvested from fields fertilized with poultry or swine manure with increased copper are other causes of poisoning [7]. Misformulation of rations or errors in the mixing of feed can result in high concentrations of copper.

Chronic copper poisoning is a common global problem for the sheep industry, and outbreaks were reported and case reports were published in many sheep-rearing countries, such as Iran, Australia, New Zealand, South Africa, USA, Brazil, and Canada [7]. In Europe, recent reports from the United Kingdom described cases of copper poisoning in sheep, associated with organic farming and the consumption of red clover (*Trifolium pratense*) and white clover (*Trifolium repens*) [11]. It is also a common problem in the Greek sheep industry [11]. Cases were also reported in Spain, Turkey, and the Scandinavian countries [7]. In Belgium, commercially prepared milk replacers with high copper levels were involved in poisoning cases in calves [11].

In this case report, we describe an episode of accidental chronic copper poisoning in Texel sheep due to feeding the animals with a compound feed containing mineral supplements intended for pigs. The mineral supplement contained copper bilysinate, a new form of copper, which was authorized in 2014 in the European Union for animal nutrition for all animal species. Also in 2014, European Food Safety Authority (EFSA) showed an equivalent bioavailability between copper bilysinate and copper sulfate [12]. This feed additive can be a powder or a granulate with a content of copper ≥ 14.5% and lysine-HCl ≥ 84%; the active substance name is copper chelate of L-lysinate-HCl, and the chemical formula is Cu(C_6_H_13_N_2_O_2_)_2_ [12]. In addition to the high copper content, according to the manufacturer, the commercial product involved in this poisoning case reduces copper excretion by 43.6% in pigs (the species for which the product is labeled) [13], which can potentially increase the risk for hepatic accumulation and chronic poisoning in sheep.

## 2. Case Description

The episode occurred in a small owner-operated unit in Cluj County, Romania, in January 2022. The farmer, who was new in sheep husbandry, incorrectly purchased feed with added copper bilysinate intended for pigs. Five animals were involved in this poisoning case: one male and four pregnant ewes, Texel breed of sheep. The animals were acquired one month before the onset of the clinical signs and were housed in a stable, where they had permanent free access to fresh water and were fed with good-quality hay and a compound feed with mineral supplement intended for pigs, containing 14.22 mg/kg copper bilysinate. There was no added molybdenum in this commercial product.

The animals started to show clinical signs 6 weeks after the introduction of the commercial compound feed into the diet. The first animal to show clinical signs was a three-year-old pregnant ewe. The animal showed anorexia, weakness, and apathy. Jaundice, characterized by the yellow discoloration of the conjunctiva and the skin, hemoglobinuria, abdominal pain, rumen stasis, and normal body temperature were observed. Although it was able to rise, the animal preferred sternal recumbency and died 2 days after the onset of the clinical signs. A week later, a second animal, a three-year-old male, started to show similar clinical signs and died. After this event, the owner suspected a feed-related problem and stopped feeding the surviving animals with the high copper level feed. The hay was examined, and no visible signs of impurities or toxic plants were identified. The animals had permanent free access to fresh water, the public water system being the source of water at this farm.

Although the compound feed was removed from the diet, after another 3 weeks (week 10 after the introduction of the compound feed), another pregnant ewe died, presenting similar clinical signs and lesions as the previous animals showed.

The two surviving pregnant ewes presented mild clinical signs of anorexia and apathy, fully recovered, and gave birth to healthy lambs.

**Figure 1:**
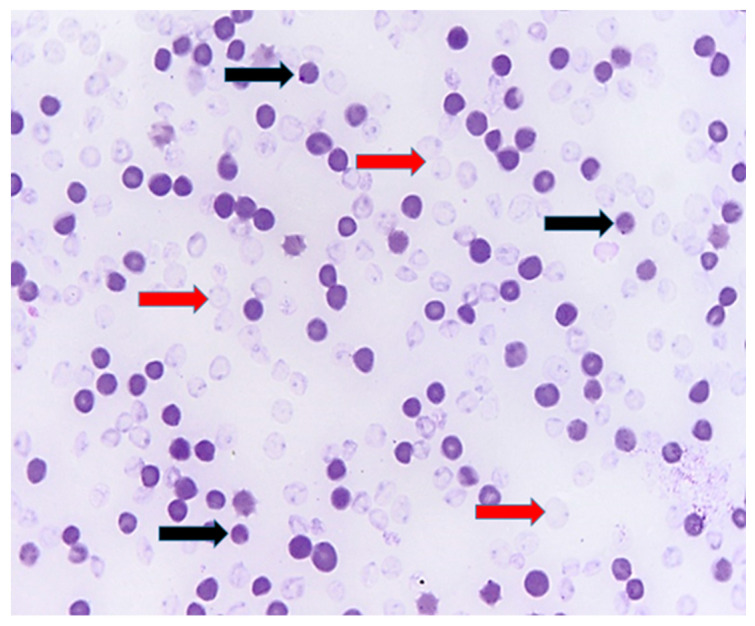
Blood smear, evidence of Heinz bodies (black arrows) and ghost erythrocytes (red arrows), indicating hemolytic anemia; Diff-Quick stain.

Blood smear realized from peripheral blood showed signs of oxidative hemolytic anemia, with the presence of erythrocyte ghosts and Heinz bodies (Figure 1).

**Figure 2:**
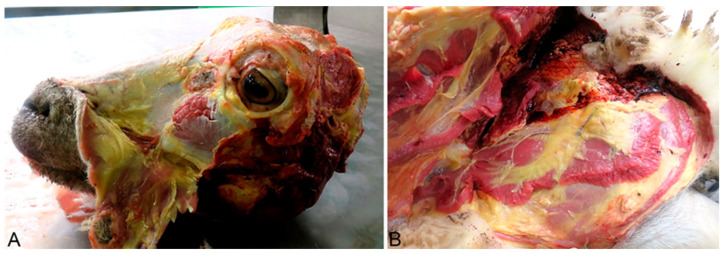
(**A**) (Head), (**B**) (Left hindlimb). Generalized icterus, yellowish discoloration of the skin and subcutaneous fat, caused by hyperbilirubinemia resulting from intravascular hemolysis.

**Figure 3:**
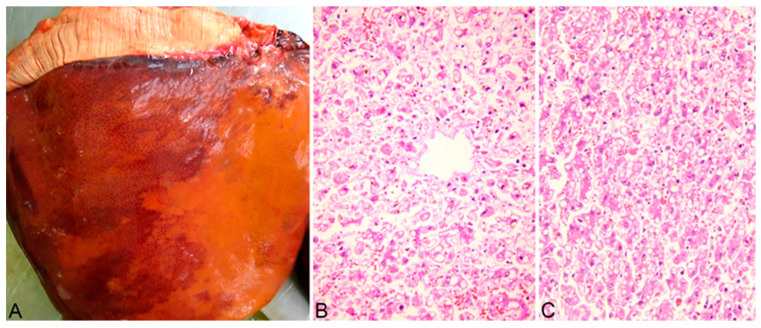
Pale, enlarged liver (**A**). Massive, diffuse hepatocellular necrosis, centrolobular area (**B**) and midzonal area (**C**). Hematoxylin-eosin stain (**B**,**C**).

**Figure 4:**
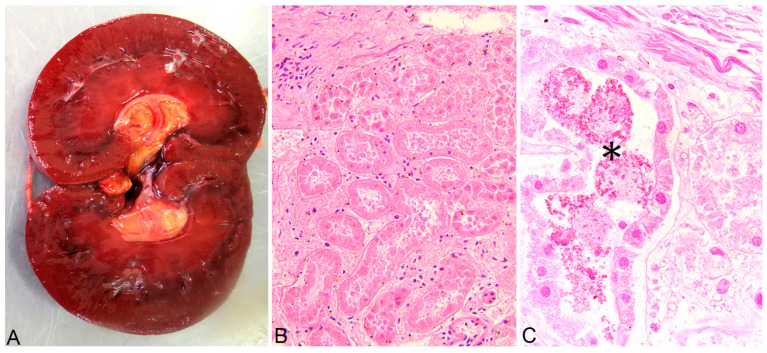
Kidney, diffuse dark-brown discoloration of the renal cortex (**A**), Acute, severe renal tubular necrosis—all tubular structures in the section are affected (**B**). Multifocal intraluminal hemoglobin casts—asterisk (**C**). Hematoxylin-eosin stain (**B**,**C**).

**Figure 5:**
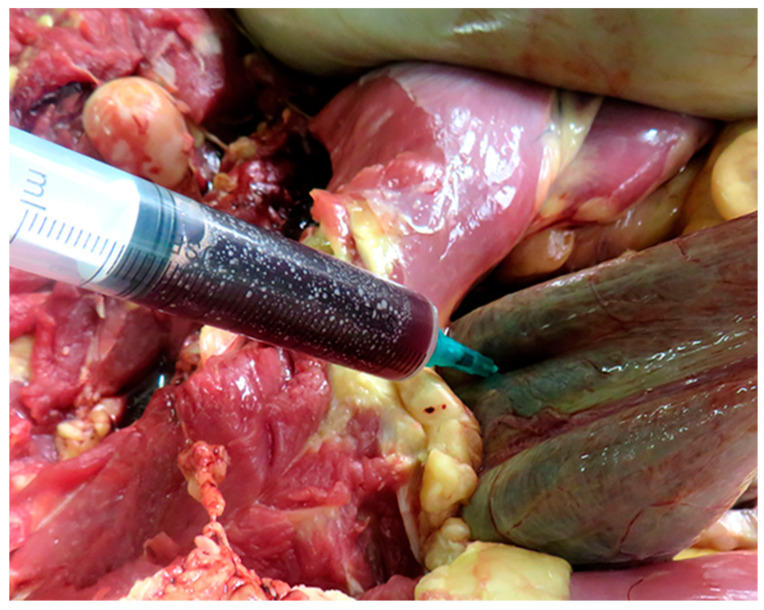
Dark-brown urine—hemoglobinuria, likely resulting from excessive intravascular hemolysis.

A complete necropsy was performed on the deceased animals, and the poisoning was confirmed through pathological findings. Grossly, the animals showed marked, diffuse icterus (Figure 2A,B), splenomegaly, pale, friable, and enlarged liver (Figure 3A), distended gallbladder, dark-brown discoloration of the renal cortex (Figure 4A), and hemoglobinuria (Figure 5).

The main histological findings were acute, massive, diffuse hepatic necrosis (Figure 3B,C) and diffuse, severe renal tubular necrosis associated with the presence of hemoglobin casts within renal tubules (Figure 4B,C).

## 3. Discussion

Copper intoxication is a deadly condition in sheep and is reported worldwide [7]. There are two types of copper toxicosis described: acute and chronic [1,7,9,14]. Copper sulfate is typically the form most frequently associated with reported cases of poisoning [9]. In sheep, especially in sensitive breeds like the Texel sheep, the chronic form is more common, and it is usually a consequence of long-term exposure (several weeks) to excessive doses (higher than the dietary requirement) [7]. The incidence of chronic copper poisoning is increasing worldwide as intensive breeding systems are extensively used and sensitive breeds become more popular [7]. The main cause of chronic copper poisoning in sheep is the inability of this species to increase biliary excretion as a response to high dietary intake [14]. High dietary intake will lead to increased absorption, as copper is not absorbed based on the animal’s daily requirements like other metals but is dependent and proportionate on the concentration in their diet [15].

In sheep, copper has a complex interrelationship with several elements, especially molybdenum and sulfur [9], as both elements decrease copper absorption and inhibit copper utilization. These minerals interact to form soluble thiomolybdates (mono, di-, tri-and tetra) in the rumen [1]. Thiomolybdates are also absorbed into the bloodstream and act as a copper chelator, keeping copper in a biologically non-functional form. Molybdenum plays a major role, with low dietary levels of the metal being associated with increased absorption of copper. Other metals and minerals that interact with copper absorption are interacting zinc and iron [15]. Interaction with the mentioned minerals, even if the dietary level of copper is within the normal range, may also lead to the development of chronic poisoning [1,7,9].

The elevated copper level in the diet (or molybdenum deficiency) leads to liver accumulation of the metal [1]. The chemical form of copper in the diet is also important as the bioavailability may vary among different products. Newer feed additives, such as copper bilysinate have high bioavailability [7]. In addition to the high bioavailability, the commercial product involved in this episode reduces copper excretion [13].

While the liver accumulates copper, the plasma and serum copper concentrations are maintained within the normal range [1]. When copper levels in the liver reach high, toxic levels, and the storing capacity of lysosomes is overwhelmed, liver necrosis occurs [14]. Copper is then released from the necrotized hepatocytes into the bloodstream, causing oxidation of the erythrocyte membrane resulting in methemoglobinemia and intravascular hemolysis, hemoglobinuria, and elevated serum levels [9,14]. The copper release from the hepatocytes is usually triggered by a stress such as transportation, pregnancy, lactation, extreme weather, and concurrent diseases [1,7,9].

The cause of death in chronic copper poisoning is either acute anemia due to hemolysis or acute renal tubular necrosis induced by the direct toxic effect of the reabsorbed copper by the renal tubular epithelial cells and by the toxic effects of hemoglobin, which is reabsorbed after the hemolytic event [7,9,14].

In the present episode of chronic copper poisoning, the diagnosis was based on the history, clinical data, and post-mortem findings. Feeding a susceptible breed with a high copper diet over several weeks led to copper accumulation in the liver and release of the metal with typical clinical signs and lesions for chronic copper intoxication. Previous studies [16] showed that copper lysine complex administration leads to higher liver concentrations compared to copper sulfate in pigs [16]. In this study, the use of a commercial compound feed containing copper bilysinate, which, according to the manufacturer, reduces copper excretion by more than 43% in the target species (pigs), led to high liver concentrations, hepatocellular necrosis and intravascular hemolysis in the sheep involved in this episode.

Breed is one of the most important factors influencing copper toxicity, and genetic factors could be involved [7]. Merino sheep are less often affected by copper toxicoses than other sheep breeds [17]. North Ronaldsay, reported to be the most susceptible breed, is a primitive sheep adapted to the copper-deprived North Ronaldsay island [7,17]. Finnsheep seems to be the most resistant [18]. A copper content of less than 15 ppm in the diet is considered safe yet it can accumulate in the liver of Texel sheep and Texel x Friesian milk sheep at a relatively high level, which suggests that the sensibility of these breeds to copper is high [18]. Breeds susceptible to copper intoxication, like Texel, along with mature British breeds, like Suffolks, Oxfords, and Shropshires, are often used in the sheep industry, increasing the incidence of the disease [6,7,18].

As the clinical signs in chronic copper poisoning are associated with an acute hemolytic crisis, the differential diagnosis should exclude several infectious diseases such as babesiosis, leptospirosis, theileriosis, anaplasmosis, bacillary hemoglobinuria, Clostridium perfringens type A infection, and toxic plant ingestion such as rape, kale, onion, garlic [7].

Animals suffering from chronic copper poisoning have a poor prognosis and can be difficult to treat when they show severe clinical signs [7] such as in the present episode, where even after the compound feed was removed, another animal died. It is therefore recommended that animals with copper poisoning are treated before the onset of severe clinical signs [9].

Treatment of chronic copper poisoning is realized by administering copper antagonists and chelating agents [7,9,14]. In animals with severe anemia following intravascular hemolysis, a blood transfusion should be considered [7,9]. The most used antagonists are ammonium or sodium molybdate and sodium thiosulphate. These products are administered as a drench, daily, orally, 50–500 mg ammonium or sodium molybdate and 0.3–1 g sodium thiosulphate. The therapy should last 3–4 weeks [7,9,14].

The most effective product in reducing liver copper levels and reducing mortality rates is ammonium tetrathiomolybdate. It is administered intravenously, at 2.7 mg/kg for 3–6 treatments (on alternate days) or subcutaneously at 3.4 mg/kg on three alternate days [7,9,14].

The chelator used in chronic copper poisoning in sheep is D-penicillamine 10 to 15 mg/kg orally twice daily [14]. Sodium calcium edetate and 2,3-dimercapto-1-propanol have also been experimentally used to treat copper poisoning in sheep with variable success [7,14].

Preventing hepatic copper accumulation and reducing the risk of chronic poisoning in susceptible breeds, especially in intensive breeding systems or endemic areas, are mainstays of ovine health management [7]. Prevention is realized with the addition of copper antagonists to the diet. Dietary supplementation with molybdenum (can be increased to 5 ppm in the diet) and sulfur (0.35% of diet) reduces hepatic Cu accumulation in sheep. Zinc supplementation at 100 ppm will also reduce copper absorption [6,14]. A copper/molybdenum ratio of 6:1 to 10:1 in the diet decreases the likelihood of copper accumulation in the liver [14].

Copper bilysinate is a relatively new, recently registered supplement. To the authors’ knowledge, this study is the first report of poisoning in sheep involving this new form of copper. In addition to exposure to this highly bioavailable copper source, other ingredients of the commercial compound feed involved in this episode may have contributed to reduced excretion of copper, finally leading to accumulation and chronic copper poisoning. It is essential not to use compound feeds intended for copper-tolerant species like pigs in the diet of sheep.

## Data Availability

The data generated in this study are presented in this article.
